# Integrative effects of dystrophin loss on metabolic function of the mdx mouse

**DOI:** 10.1038/s41598-018-31753-3

**Published:** 2018-09-11

**Authors:** Jana Strakova, Forum Kamdar, Debra Kulhanek, Maria Razzoli, Daniel J. Garry, James M. Ervasti, Alessandro Bartolomucci, DeWayne Townsend

**Affiliations:** 1 Department of Integrative Biology and Physiology, Medical School, University of Minnesota, Minneapolis, MN USA; 2 Department of Medicine, Cardiovascular Division, Medical School, University of Minnesota, Minneapolis, MN USA; 3 Department of Biochemistry, Molecular Biology, and Biophysics, Medical School, University of Minnesota, Minneapolis, MN USA

## Abstract

Duchenne muscular dystrophy (DMD) is a disease marked by the development of skeletal muscle weakness and wasting. DMD results from mutations in the gene for the cytoskeletal protein dystrophin. The loss of dystrophin expression is not limited to muscle weakness but has multiple systemic consequences. Managing the nutritional requirements is an important aspect of the clinical care of DMD patients and is complicated by the poor understanding of the role of dystrophin, and dystrophic processes, in regulating metabolism. Here, we show that mdx mice, a genetic model of DMD, have significantly reduced fat mass relative to wild type C57BL/10. The alteration in body composition is independent of the presence of skeletal muscle disease, as it is still present in mice with transgenic expression of a fully-functional dystrophin in skeletal muscle. Furthermore, mdx mice do not increase their fat mass or body weight when housed under thermoneutral conditions, in marked contrast to C57BL/10 mice. We also demonstrated that mdx mice have significantly reduced fat metabolism and altered glucose uptake. These significant metabolic changes in dystrophic mice implicate dystrophin as an important regulator of metabolism. Understanding the metabolic functions of dystrophin is important for managing the nutritional needs of DMD patients.

## Introduction

Duchenne muscular dystrophy (DMD) is a devastating disease resulting from the loss of the protein dystrophin^1^. Dystrophin is expressed in the greatest amounts in striated muscles, but is expressed at lower levels in many tissues^2^. In striated muscles, dystrophin appears to play a major role in the protection of the sarcolemmal membrane from the forces of contraction^3^. The role of dystrophin in non-muscle tissues is less clear. Dystrophin binds to a wide variety of proteins within cells, it’s interactions with dystroglycan and the sarcoglycan complex are thought to mediate dystrophin’s mechanical protection^4,5^. Dystrophin also binds to many other proteins forming a nidus of a signaling complex, this later function is thought to be critical function of dystrophin expression in non-muscle cells^6,7^.

The disease course of DMD is first evident as skeletal muscle weakness during the first five years of life. There is a progressive loss of skeletal muscle, which is replaced with fatty fibrotic tissue. The disease continues to progress with eventual respiratory and/or cardiac dysfunction eventually leading to death during the third or fourth decade of life. While respiratory and cardiac disease are the most common causes of mortality, the proper management of nutritional status is an important aspect maintaining the quality of life in DMD patients and their care givers^8^. This is particularly challenging in DMD patients who face various metabolic challenges during the course of the disease. Early in the course of the disease patients often receive steroids to prolong ambulation^9^, however these drugs significantly alter metabolic function, including an increase in obesity. With the loss of ambulation, energy expenditure is markedly reduced which further increases the risk for obesity. As the disease progresses further, skeletal muscle continues to be lost, which can result in weight loss and under-nutrition^10^. Evaluating DMD patients for obesity can be difficult as standard measures of obesity, such as body mass index and body composition often require invasive measures which are difficult in DMD patients^8^.

Further complicating these issues are the changes in body composition that are uniquely linked to the progression of DMD. Patients with DMD display an increase in fat mass that is positively correlated with age and, conversely, lean tissue mass is negatively correlated with age^11,12^. The loss of lean mass is consistent with the dystrophic process and the increase in adiposity could result from the decreased activity associated with the loss of ambulation as well as the steroid treatment. However, the unique distribution of this fat mass suggests that the picture is more complicated. There is no difference in visceral or subcutaneous adiposity, which would be expected if the increased fat mass occurred secondary to changes in energy expenditure. Interestingly, most of the increased fat mass result from ectopic fat accumulating in muscles, with 8 times more intramuscular adipose tissue than healthy controls^13^. These data suggest that the age-related increase in fat mass is secondary to the fat replacement of dystrophic muscle, which raises questions about the role of this fat in the overall metabolic picture in DMD patients. This model is further supported by the age correlated loss of lean muscle mass in limbs of DMD patients^14^.

Skeletal muscle is well defined as a critical metabolic organ having important roles in energy expenditure and glucose metabolism. Previous studies in dystrophic mice have demonstrated reductions in fat mass and alterations of metabolic activity^15–19^. To better understand the metabolic consequences of the absence of dystrophin we extend the metabolic assessment of the mdx mouse and address the role of muscle pathology using transgenic mdx mice.

## Results

### Dystrophin deficiency results in a lean phenotype

**Figure 1:**
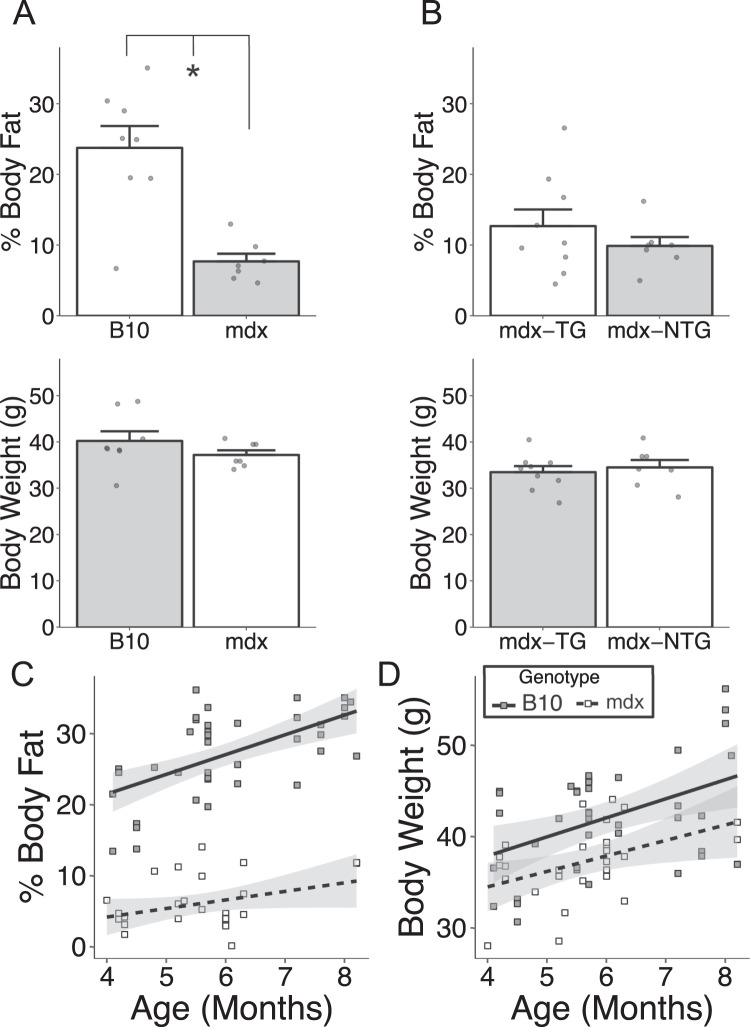
Global loss of dystrophin is associated with a lean body composition. (**A**) Mice lacking dystrophin (mdx) have a significant reduction in body fat relative to their dystrophin replete littermates. B10 age: 4.8 ± 0.2 months, mdx age: 4.8 ± 0.2 months. (**B**) Skeletal muscle specific transgenic expression (mdx-TG) of a C-terminally truncated dystrophin does not have a significant impact on body composition relative to non-transgenic mdx mice (mdx-NTG). (**C**) Regression plot demonstrating attenuated of body fat accumulation in mdx mice with age, although body weight increases are similar (**D**). Mdx-TG age: 6.3 ± 0.2 months, mdx-NTG age: 6.2 ± 0.2 months. *P < 0.001, error bars (panels A and B) represent SEM, shaded regions in panels C and D represent 95% confidence intervals.

We observed that dystrophin deficient male mice have markedly reduced body fat mass compared to their dystrophin replete siblings (Fig. 1A), with no difference in body weights. This striking difference in body composition is also present between female B10 and mdx (30.3 ± 2.9 vs. 10.3 ± 0.9% body fat, respectively, P < 0.0001). To extend our observations, we analyzed another mouse model of muscular dystrophy resulting from deficiency in a dystrophin-associated protein, i.e. β-sarcoglycan (β-SG)^20,21^. *β-SG* deficient mice display a similar level of central nucleation and cellular infiltration within the skeletal muscle as that observed in mdx mice, but retain normal levels of dystrophin^22^. Compared to C57BL/6 mice, the *β-SG*-null mice did not have significant changes in body mass or body composition (Supplemental Fig. 1), indicating that dystrophy alone is not sufficient to alter body composition. It is also clear that there are significant differences in the body composition of mice on the C57BL/10 and C57BL/6 genetic backgrounds. The rest of the studies will focus on mice from only a C57BL/10 genetic background.

Age is another important factor contributing to body composition. Linear regression analysis demonstrates that wild type C57BL/10 mice display a significant increase in % body fat of 2.8 ± 0.6% body fat/month (Fig. 1C). In contrast, aging mdx mice fail to accumulate fat to the same degree (1.2 ± 0.7% body fat/month). This is in marked contrast to body weight measures which both C57BL/10 and mdx mice gain body weight with age (Fig. 1D).

To explore the mechanism by which the absence of dystrophin may result in metabolic changes leading to alterations in body composition, a transgenic mdx mouse with skeletal muscle specific expression of a C-terminally truncated dystrophin was used. This transgenic mouse has fully corrected skeletal muscle pathology and has been extensively backcrossed onto the mdx genetic background^23,24^. These mice demonstrated the same lean body composition, similar to the mdx mouse, regardless of the presence of the transgene (Fig. 1C). These data suggest, surprisingly, that the presence of skeletal muscle pathology in dystrophic mice does not contribute to the lean body phenotype in mice lacking dystrophin.

### Effects of altering metabolic demands on body composition of dystrophic mice

**Figure 2:**
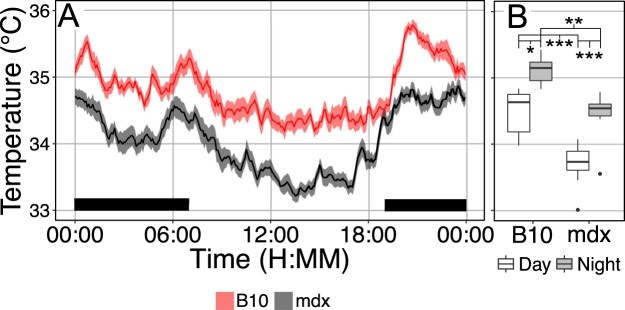
Core body temperature is significantly reduced in mdx mice. Telemetry derived core body temperature are shown. (**A**) Core body temperature median and interquartile range from C57BL/10 and mdx mice both during the day and during the night (black bar). (**B**) Boxplots of mean daily and nightly core body temperatures in dystrophic mice. Data are derived from 8 C57BL/10 (B10; 4.4 ± 0.4 months) and 8 mdx mice (6.0 ± 0.3 months). For each mouse data are averaged from three days of observations, data in (**A**) is mean ± SEM. *P < 0.05, **P < 0.005, ***P < 0.001.

**Figure 3:**
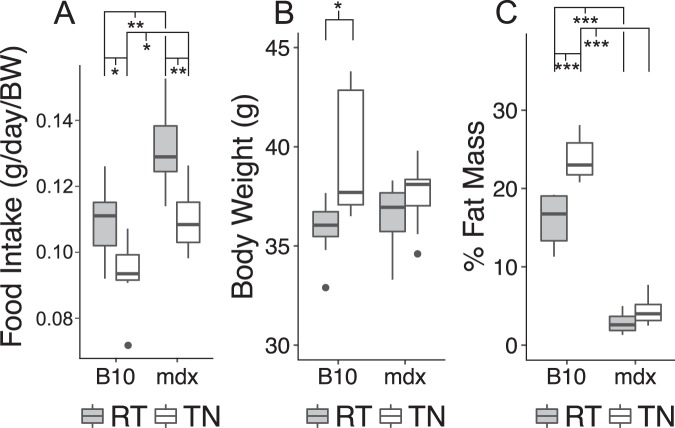
The effects of thermoneutral housing on C57BL/10 and mdx mice. (**A**) Daily food consumption, normalized to body weight, is significantly greater in dystrophic mice both at room temperature (RT) and thermoneutral (30 °C) temperature (TN) for 30 days. Both strains of mice display significant declines in food intake with thermoneutral housing. In C57BL/10 significant increases in body weight (**B**) and fat mass (**C**) occur with thermoneutral housing. In contrast, mdx mice have no change in body weight or body composition with thermoneutral housing. Data based on group size of 8 mice per group; B10 age: 5.3 ± 0.1 months and mdx age: 5.1 ± 0.1 months. *P < 0.05, **P < 0.01, ***P < 0.00001.

Radio-telemetry studies revealed that mdx mice have consistently lower core body temperatures compared to C57BL/10 mice (Fig. 2). Attempts of the mdx mouse to maintain body temperature may represent a significant metabolic stress, that may contribute to the lean body composition observed in mdx mice. The temperature of thermoneutrality for mice is ~28–30 °C. Housing below this temperature leads to rapid heat loss to the environment which needs to be compensated by increased energy expenditure to defend their core body temperature^25,26^. If mdx mice maintain a lean body composition because of increased metabolic demand or limitations on food intake, housing under thermoneutral conditions should reduce the basal metabolic requirements allowing a normalization of the lean phenotype. To test this hypothesis, the body composition and food intake of a cohort of C57BL/10 and mdx mice were measured during room temperature housing followed by similar measurements with housing under thermoneutral conditions. Compared to housing at room temperature both C57BL/10 and mdx mice ate significantly less food at thermoneutrality, consistent with the removal of the metabolic demands of maintaining core body temperature (Fig. 3). Interestingly, under both room temperature and thermoneutral conditions mdx mice ate significantly more food per gram of body weight compared to C57BL/10 mice. As expected, C57BL/10 mice gained body weight and increased adiposity with exposure to thermoneutrality. In contrast, mdx mice had no change in body weight or body composition under thermoneutral conditions suggesting sustained energy expenditure in spite of the housing at thermoneutrality. These data indicate that the lean body composition of the mdx mouse was not related to reductions in food intake, which is higher in mdx mice, nor can it be eliminated by minimizing thermogenic demand by housing at thermoneutrality.

### Dystrophic mice are hypermetabolic

**Figure 4:**
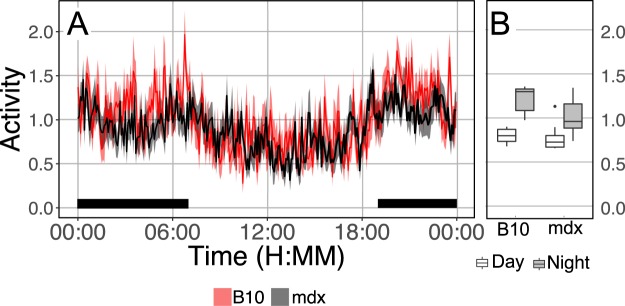
Telemetry based measurement of activity in wild type and dystrophic mice. There is no significant difference in the activity measurement between wild type and dystrophic mice. Both strains display significant increases in activity during the night (black bar) relative to the light period. Data are derived from 8 C57BL/10 (B10; 4.4 ± 0.4 months) and 8 mdx mice (6.0 ± 0.3 months). Data displayed in time series represents mean ± SEM.

Given the observation that reducing thermogenic requirements had no impact on the lean body composition of mdx mice, the hypothesis that increased metabolic activity contributes to the lean body composition of dystrophic mice was tested using indirect calorimetry. The rate of oxygen consumption is based on many factors^27–29^. Activity is a prominent driver of oxygen consumption, as seen by the significant increase in oxygen consumption during nightly activity^26^. To assess the importance of potential differences in activity we used both home cage telemetry-derived activity (Fig. 4) and beam break activity assessments in the indirect calorimetry chamber. In both of these measures there is no significant difference in activity between C57BL/10 and mdx mice.

**Figure 5:**
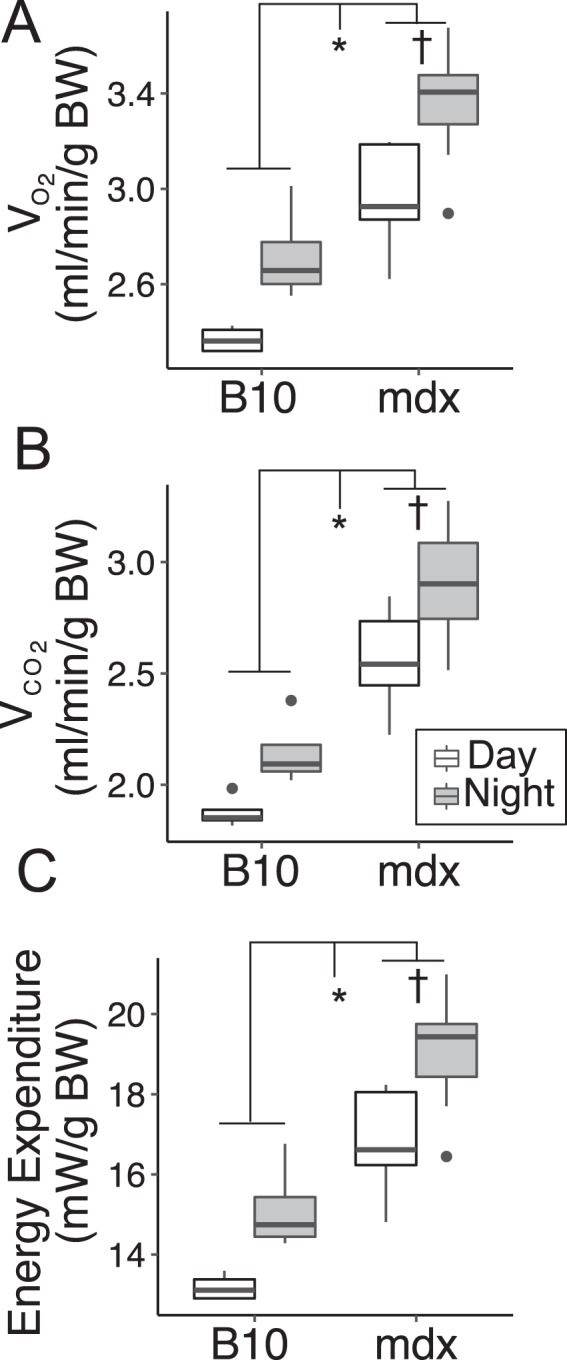
Results from indirect calorimetry of C57BL/10 and mdx mice. (**A**) Oxygen consumption, (**B**) carbon dioxide production, and (**C**) energy expenditure normalized to body weight (BW). Time of day is a significant determinant of these parameters and this data is depicted as day (white) and night (grey). Data represents mean values from 30-hour period from 7 mdx (5.8 ± 0.3 months) and 4 C57BL/10 mice (5.3 ± 0.3 months). *P < 0.001 genotype effect, ^†^P < 0.01 time of day effect.

Another important component driving oxygen consumption, carbon dioxide production, and energy expenditure is the mass of the animal. Correction for total body mass demonstrates that mdx mice consume more oxygen, produce more carbon dioxide, and have higher energy expenditure compared to C57BL/10 mice (Fig. 5) per gram of body weight. The elevated energy expenditure per gram of body weight in the mdx mouse is consistent with the increase in food consumption, as caloric intake closely matches energy expenditure.

### Respiratory exchange ratios are significantly higher in dystrophic mice

**Figure 6:**
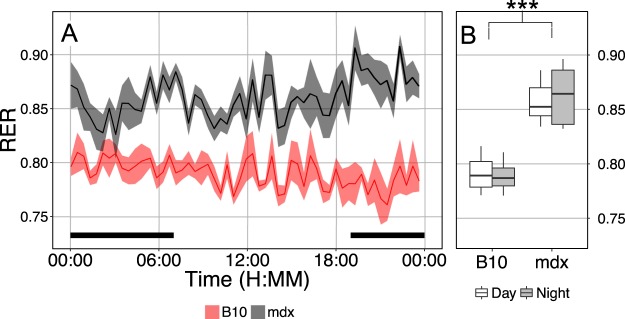
Respiratory exchange ratio (RER) derived from indirect calorimetry in C57BL/10 (B10) and mdx mice. (**A**) Data represent the mean ± SEM of all RER measurements collected from 4 C57BL/10 and 7 mdx mice during a 30-hour measurement period. (**B**) Boxplot displaying the average daily (white) and nightly (grey) RER measurements from the same mice in panel A. ***P < 0.00001 genotype effect, there is no significant effect of the time of day on RER.

**Figure 7:**
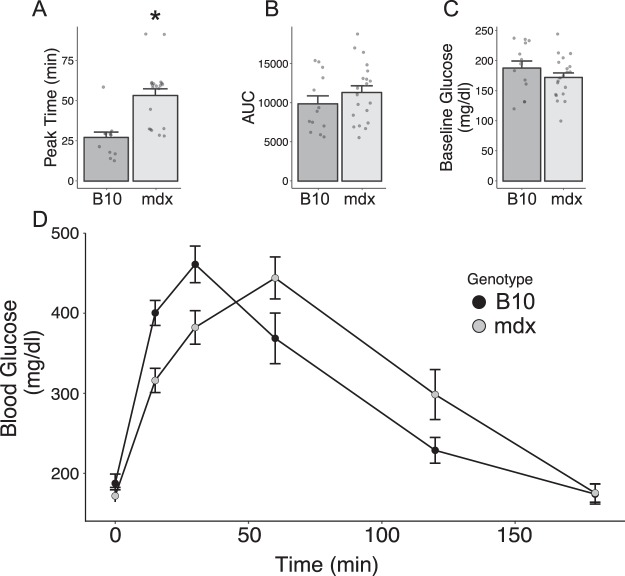
Glucose tolerance test in C57BL/10 and mdx mice. (**A**) The peak of glucose uptake is significantly delayed in mdx mice relative to wild type mice. There are no significant differences in the area under curve (AUC) between wild type and mdx (**B**). There are no significant differences in baseline glucose levels (**C**). Aggregated glucose levels present as means ± SEM (**D**). Data from 13 C57BL/10 (B10; 5.0 ± 0.2 months) and 20 mdx (5.8 ± 0.2 months) mice *P < 0.05 vs. B10.

Of the significant genotype differences observed, carbon dioxide production in the dystrophic mouse displays a greater difference than the increased oxygen consumption resulting in a significant increase in the respiratory exchange ratio (RER) in dystrophic mice (Fig. 6). The higher RER observed in mdx mice of 0.86 ± 0.01 indicates a lower level of lipid metabolism^29^ when compared to B10, likely driven by decreased supply of lipid substrates in mdx mice. To further assess the potential for differences in carbohydrate metabolism, glucose tolerance tests (GTT) were performed in C57BL/10 and mdx mice (Fig. 7). This carbohydrate challenge revealed that mdx mice have a delay in the peak glucose concentration, suggesting that initial glucose uptake was faster in the dystrophic mice (Fig. 7). However, the overall area under the curve (AUC) is not significantly different from that observed in wildtype mice, suggesting that overall glucose uptake is not significantly altered in dystrophic mice.

## Discussion

The role of dystrophin in the regulation of metabolism is poorly understood. This results from the multi-systemic nature of the disease that results from the loss of this protein. Most notably the loss of dystrophin results in significant dysfunction of skeletal muscle, an important organ regulating systemic metabolism. Clinically, nutritional management of patients with muscular dystrophy is complicated by many factors including therapeutic steroids, decreased activity, muscle wasting, and difficulty obtaining sufficient calories. The current study suggests that the loss of dystrophin alone may have direct impact on the metabolism of DMD patients.

It must be noted that the studies reported here rely on the mdx mouse model of DMD and the correction of the increased body fat phenotype observed in C57BL/10 (the background strain of the mdx mouse) compared to the more commonly studies C57BL/6. The underlying genetic cause of this obesity is unknown, which complicates the interpretation of the data presented in the current study. However, it is clearly demonstrated that the absence of dystrophin is closely correlated with the loss of the high fat phenotype in the C57BL/10 mouse. Furthermore, this study and others^15,17,18^ demonstrate that mdx mice are unable to add fat even under conditions where that would be expected (i.e. high fat diet or thermoneutral housing). Interestingly, this all occurs in spite of mdx mice consuming more food than C57BL/10 counterparts (Fig. 3). It is possible that mdx mice have limited apidogenic precursors and thus fewer adipocytes, but this would be expected to result in large increases in plasma lipids. Studies examining serum lipid levels in mdx mice find no significant difference from wild type mice^18^. Together, these data suggest that the lean body phenotype of mdx mice results from decreased storage of lipids by adipocytes and not increased lipolysis.

The transgenic mice used in this study have been extensively backcrossed (>10 generations) on to the C57BL/10 background strain^23^, yet these mice, regardless of genotype retain a lean phenotype. These mice, which have largely corrected the dystrophic process in skeletal muscle, suggest that the lean phenotype is not dependent on the presence of muscle pathology. It is possible that the expression of a C-terminally truncated dystrophin may not provide a critical functional domain to facilitate the high-fat phenotype seen in wild type C57BL/10 mice expressing full-length dystrophin. Another intriguing possibility is that expression of dystrophin in non-skeletal muscle tissue may have an important metabolic function. Specifically, dystrophin and components of the dystrophin-associated glycoprotein complex have been identified in adipose tissue by western blotting^30,31^. This observation raises the possibility that the dystrophin related effects may lie within the adipose tissue. This would be consistent with the observations that dystrophic mice fail to increase adipose tissue mass with high-fat diets^15^ or with thermoneutral housing conditions (Fig. 3).

The importance of these observations in DMD patients remains to be directly addressed and is complicated by the metabolic effects of the steroids used to treat this disease. Managing the metabolic state of dystrophic patients is challenging and many factors contribute to changes in body composition. As DMD progresses, patients demonstrate declines in lean body mass and increases in fat accumulation^11,12,32^. Initially this appears in direct contrast to what is observed in the mice; however, the localization of this new fatty tissue is of interest. Age related increases in fat tissue in DMD patients are driven by increases in ectopic fat mass within deteriorating skeletal muscle^13,33^, there is very little change in subcutaneous fat. This is further supported by evidence that patients with the greatest strength have the least amount of fat accumulation within their muscle tissue^32^. This raises the intriguing possibility that the presence of intra-muscular fat may contribute to the disease process and the absence of this in the mdx mouse might contribute to the overall mild phenotype observed in this model.

In these studies, we observed that mdx mice have an increased energy expenditure per gram of body weight relative to C57BL/10 mice, this may result from lean mass generally contributing more than fat mass to the total energy expenditure^28,34^. Increases in fat-free tissue mass have been shown to be correlated with higher resting energy expenditure^35^, the hypothesis being that leaner tissues consume more oxygen relative to tissues with increased fat content. This observation is consistent with previous results examining energy expenditure in mdx mice^16^. The hypothermic phenotype observed in the mdx mice appears in contrast to the increased energy expenditure and an intriguing possibility that it may be a driving force to increase energy expenditure, leading to increased food consumption. It is possible that mdx mice are not able to consume enough food to meet the metabolic demands of continuous muscle regeneration, resulting in an inability to maintain normal body temperature. However, such an explanation would expect to result in significant reduction in overall growth, which is not observed. Another possibility is that the absence of dystrophin may affect the thermoregulatory set point through actions within the brain.

The delay in the peak glucose level following a bolus injection can be interpreted as a rapid early uptake of glucose in mdx mice, consistent with the observation that GLUT4 expression is increased in mdx skeletal muscle^36^. Stapleton *et al*. demonstrate increased levels of muscle glycogen, but reduced levels of liver glycogen in mdx mice relative to C57BL/10^37^. These studies also observe significant elevations in RER, which suggest high levels of carbohydrate metabolism can also occur with high levels of protein catabolism (31). There is significant evidence that both dystrophic patients^38^ and mice^16^ have increased protein catabolism. The energy expenditure derived from indirect calorimetry of the mdx mouse is 17.6 ± 0.5 mW/g_BW_, which is in good agreement with total energy intake of 19.1 ± 0.6 mW/g_BW_. The standard equation for energy expenditure usually considers the contribution of protein catabolism to be negligible^28,39^. In fact, not correcting for differences in protein oxidation induces only a small error, unless protein oxidation exceeds 15% or total energy expenditure^29^. The strong correlation between energy expenditure and energy intake suggests that protein oxidation does not contribute significantly to overall metabolic flux in dystrophic mice. These data are consistent with a process by which muscle protein turnover is quite high, but the amino acids are quickly incorporated into new proteins rather than oxidized for energy production. Importantly, these changes in protein metabolism is not likely mechanistically linked to the lean phenotype observed in the mdx mouse, given that it is independent of muscle pathology.

Managing the metabolic complications of DMD will become increasingly important as new therapies continue to extend the life expectancy. Understanding dystrophin’s functions outside of striated muscle will also be important for the development of new therapeutic approaches. These studies confirm that the dystrophic mouse can be a valuable tool in understanding the role of dystrophin in modulating metabolic function.

## Methods

### Animals

The mice used in these studies were obtained from colonies of C57BL/10SnJ (B10) and C57BL/10ScSn-Dmd^mdx^/J (mdx) maintained at the University of Minnesota. Colony founders were obtained from The Jackson Laboratory and genetic stability ensured by the introduction of newly purchased breeding stock every 4–5 generations. The mdx and B10 mice in Fig. 1 are siblings of a cross between heterozygous mdx-B10 female and a B10 sire. Half of the males from this breeding strategy contained the mdx allele in the dystrophin locus and the other half have the C57BL/10 dystrophin allele. Genotypes of these crosses were determined by Sanger sequencing of the region containing the mdx mutation (Forward: AACTCATCAAATATGCGTGTTAGT, Reverse: CTCAATCTCTTCAAATTCTG). Transgenic mice expressing a C-terminally truncated dystrophin using the human skeletal actin promoter were originally described by Crawford *et al*.^24^ and crossed back onto the mdx background for over 10 generations^23^. These transgenic mice were genotyped using the primers: CGATCCGGTTTAGAGCAGAAGTAA and ATTCCTAGGCTCACCTCACAGG. The β-sarcoglycan knockout were originally described by Durbeej and colleagues^22^, founders were obtained from The Jackson Laboratory (B6.129-Sgcb^tm1Kcam^/1J) and bred at homozygosity in a colony at the University of Minnesota. Control C57BL/6J mice were purchased from The Jackson Laboratory. All mice in these studies were given ad libitum access to standard mouse chow (Teklad 2019, Envigo, Indianapolis, IN). All experimental protocols were preformed using methodologies reviewed and approved by the University of Minnesota Institutional Animal Care and Use Committee.

### Metabolic Phenotyping

#### Echo/MRI

Mouse body composition was measured with Echo MRI 3-in-1 (EchoMRI-900TM; Echo Medical System, Houston, TX). MRI measurements were performed in conscious mice placed in a thin-walled glass cylinder, with a cylindrical plastic insert added to limit movement of the mice to measure fat mass, fat-free (lean) mass, free water, and total water.

#### Indirect Calorimetry

Oxygen consumption (VO_2_) and carbon dioxide production (VCO_2_) were measured across 3 days using the Oxymax Comprehensive Lab Animal Monitoring System (Columbus Instruments). Energy expenditure was estimated from the ratio of carbon dioxide production and oxygen consumption^40^. The resulting data was analyzed using an ANOVA model for oxygen consumption, carbon dioxide production, and energy expenditure using genotype and time of day as a categorical predictor and total body weight and fat-free tissue mass as normalizing factors. Analysis was performed in R^41^.

#### Glucose Tolerance

Mice were fasted for 6 h, beginning at 8:00 am. A 2 g/kg D-glucose (Sigma/Aldrich) solution in sterile saline was administered i.p. and plasma glucose levels were then measured at 0, 15, 30, 60, 120, and 180 min after glucose load with a glucometer (Accucheck Aviva, Roche).The blood glucose data collected during each GTT was fit using the loess method using R^41^. The resulting fit was used to calculate time to peak glucose and total area under the curve (AUC) for each GTT performed.

#### Thermoneutrality

Body weight and food intake were monitored for 30 days in a cohort of mice while housed at room temperature. Body composition was measured at the end of this 30-day period. These mice were then housed at 30 °C for 30 days. Food intake and body weight were measured throughout this period and body composition was measured again at the end of the 30 days at thermoneutrality.

### Telemetry Data

Radio telemetry transducers (HD-X11 from DSI, St. Paul, MN) were implanted subcutaneously. Mice were allowed 1 week to recover from surgery prior to recording data. Temperature and activity data are presented here. For each individual mouse, data were smoothed by applying a 5-minute rolling median filter, this median was then averaged across three days. The data presented are the mean ± SEM for each of the 5-minute intervals from all of the mice in each genotype. Data were collected and analyzed using custom scripts in Python (data management and organization) and R (data aggregation and statistical analysis).

## Electronic supplementary material

Supplemental Figure 1
