# Supplementary material for: Integrative effects of dystrophin loss on metabolic function of the mdx mouse
Source: Sci Rep. 2018 Sep 11;8:13624. doi: 10.1038/s41598-018-31753-3 (PMC6134145; doi:10.1038/s41598-018-31753-3)
Supplement: Supplementary file 1 — Supplemental Figure 1 [file 41598_2018_31753_MOESM1_ESM.pdf]

Integrative effects of dystrophin loss on metabolic function of the mdx mouse.

Jana Strakova[1], Forum Kamdar[2], Debra Kulhanek[2], Maria Razzoli[1], Daniel J. Garry[2], James M. Ervasti[3], Alessandro Bartolomucci[1], DeWayne Townsend[1§]

[1] Department of Integrative Biology and Physiology
Medical School
University of Minnesota
Minneapolis, MN

[2] Department of Medicine
Cardiovascular Division
Medical School
University of Minnesota
Minneapolis, MN

[3] Department of Biochemistry, Molecular Biology, and Biophysics
Medical School
University of Minnesota
Minneapolis, MN

§-Corresponding Author:
DeWayne Townsend
town0045@umn.edu
3-140 CCRB
2231 6th Street SE
Minneapolis, MN 55455

Short Title: Metabolic function of the mdx mouse.

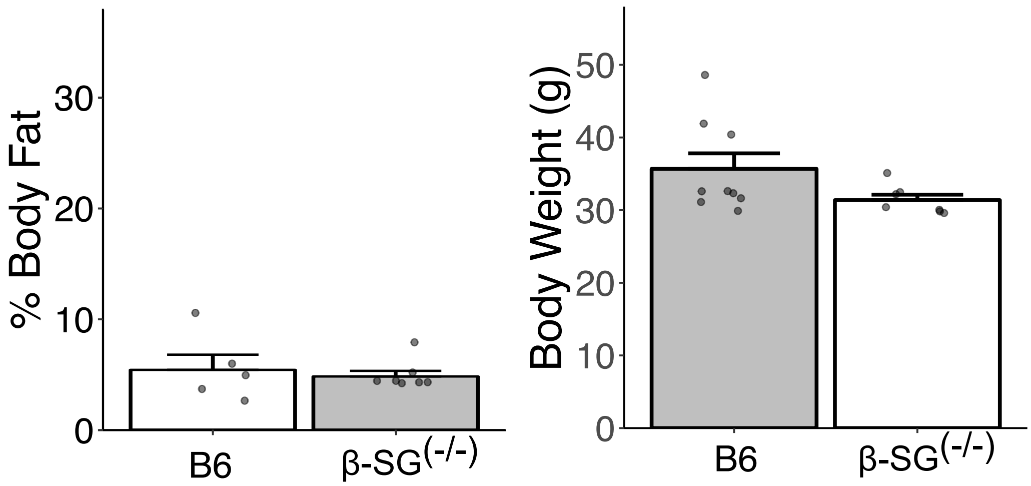

Supplemental Figure 1. Dystrophic mice lacking β-sarcoglycan (β-SG) have a lean body composition that is not significantly different from their background strain C57BL/6 (B6). B6 age: 4.7 ± 0.0 months, β-SG age: 4.9 ± 0.1 months
